# Impact of practice changes on catheter-related exit-site and bloodstream infection rates in a Canadian hemodialysis center: A retrospective study

**DOI:** 10.1177/11297298241309535

**Published:** 2025-01-24

**Authors:** Courtney K Lawrence, Michelle L Boyce, Stephanie Weisensel, Chris Sathianathan, Mauro Verrelli, Sheryl A Zelenitsky

**Affiliations:** 1 College of Pharmacy, Rady Faculty of Health Sciences, University of Manitoba, Winnipeg, MB, Canada; 2 Department of Pharmacy, St. Boniface Hospital, Winnipeg, MB, Canada; 3 Department of Pharmacy, Health Science Centre, Winnipeg, MB, Canada; 4 Max Rady College of Medicine, Rady Faculty of Health Sciences, University of Manitoba, Winnipeg, MB, Canada; 5 Manitoba Renal Program, St. Boniface Hospital, Winnipeg, MB, Canada

**Keywords:** Hemodialysis, catheter-related infection, exit-site infection, bloodstream infection, infection control

## Abstract

**Background:**

Hemodialysis vascular access predisposes patients to exit-site infections (ESIs) and bloodstream infections (BSIs), resulting in significant morbidity and mortality. The objective was to characterize hemodialysis catheter-related (CR) ESIs and BSIs while considering potential factors associated with infection.

**Methods:**

The study period was selected to coincide with new CR-infection prevention measures at the midpoint. These included masking during exit-site care, using chlorhexidine-alcohol versus povidone-iodine antiseptic, administering cefazolin prophylaxis with central venous catheter (CVC) insertions, and reducing temporary CVC use for chronic hemodialysis starts. Data were collected retrospectively, including patient characteristics, hemodialysis history, CVC details, and CR-infections. Quarterly infection rates were calculated per 1000 CVC days, and potential factors associated with infection were investigated. Modeling was used to characterize infection rates and covariates over time.

**Results:**

Over 39 months, data for 267 patients, 499 CVCs, and 114,825 CVC days were captured. During the study period, there were 113 ESIs and 64 BSIs, with >80% of infections caused by gram-positive bacteria. ESI and BSI rates were 0.98 and 0.56 per 1000 CVC days, respectively. There were significant reductions in infection rates over time. The ESI rate dropped when new CR-infection prevention measures were introduced (*p* < 0.01), from a mean of 1.28 to 0.73 per 1000 CVC days (*p* = 0.003). The rate of BSI trended downward to a low of 0.10 per 1000 CVC days in the last quarter of the study. The BSI rates associated with temporary and permanent CVCs were 1.25 and 0.53 per 1000 CVC days, respectively (*p* = 0.1). There was a strong correlation between the declining BSI rates and declining temporary CVC use over time (rho = 0.73, *p* = 0.005).

**Conclusions:**

CR-ESI rates dropped significantly when new hemodialysis CR-infection prevention measures were introduced. CR-BSI rates declined over the study period, as did the use of temporary CVCs.

## Introduction

In patients receiving hemodialysis (HD), infection is the second leading cause of mortality.^1^ The population is particularly susceptible to infections due to immunosuppression caused by end-stage renal disease (ESRD), comorbidities such as diabetes, frequent exposure to healthcare settings, and, most importantly, the need for vascular access.^2–6^ Access through arteriovenous fistulas or grafts is preferred over central venous catheters (CVCs), which are more prone to complications, including infection.^2,7–11^ Nevertheless, CVCs are an important option that may be more feasible, preferred by the patient, or required to start dialysis urgently.^10,11^

CVCs predispose patients to both catheter-related (CR) exit-site infections (ESIs) and bloodstream infections (BSIs). While less severe, CR-ESIs are a notable risk factor and source of bacteremia in patients receiving HD.^2^ The microbiology of CR-ESIs and CR-BSIs are similar, with gram-positive skin flora (i.e. staphylococci) accounting for 60%–80% of infections.^7,12^ The etiology of BSIs differs from other patient populations, where gram-negative bacteria (e.g. *Escherichia coli*) from urinary or gastrointestinal sources are more common.^13^ CR-BSIs often require CVC replacement and are associated with considerable morbidity, mortality, and healthcare costs.^14^

The published rates of CR-infections are variable due to differences in patient populations, vascular access type, infection control, and reporting criteria. Furthermore, while most investigations focus on BSIs, there are limited data on ESIs. Studies of patients receiving HD in North America, Australia, and Europe between 1999 and 2015 report CR-ESI rates of 0.46–0.80 per 1000 CVC days^7,12^ and CR-BSI rates of 0.26–2.26 per 1000 CVC days.^7,8,12,15–20^ Infection control practices are essential to prevent CR-infections in the HD population. A recent systematic review by Lazarus et al. showed that over 80% of interventional quality improvement studies to prevent CR-BSIs had favorable outcomes.^21^ More specifically, CR-infection prevention measures such as aseptic CVC insertion,^11,22,23^ antimicrobial prophylaxis with CVC insertion,^24^ aseptic techniques during catheter manipulations,^22,23^ and exit-site care^11,12,22,23^ have been associated with lower infection rates.^8,9,16^

The surveillance of CR-infections in patients receiving HD is vital to monitor infection rates, assess the effectiveness of infection control measures, and optimize the selection of empiric antimicrobial therapies. Whereas programs such as the National Healthcare Safety Network (NHSN) Dialysis Event Surveillance and ESRD Quality Incentive Program are available to support dialysis-related infection control in the United States,^9,18^ coordinated efforts and access to longitudinal surveillance data in Canada are limited. The objective of the current study was to characterize the microbiology and rates of CR-ESI and CR-BSI over 39 months in a Canadian HD center while considering potential factors associated with infection. These data were presented at the 31st European Congress of Clinical Microbiology and Infectious Diseases, July 2021 (online), Abstract #2773 (328945).

## Methods

### Study design and setting

A retrospective observational study of CR-infections was conducted in adult patients receiving HD through a CVC at St. Boniface Hospital (SBH) in Winnipeg, Manitoba. Study approvals were granted by the University of Manitoba Health Research Ethics Board (#HS20491) and the SBH Research Review Committee (#2017/1643).

The 39-month study period was selected so the midpoint coincided with the introduction of new CR-infection prevention measures. At the time, the HD unit served 145–160 patients, with 60%–70% receiving dialysis through a CVC. CVC insertions were performed by interventional radiologists or nephrologists using guidewires, ultrasound, fluoroscopy, and contrast (radiologists only) to access the vessel and verify placement. If required, CVCs were replaced at a new site, that is, not exchanged over a guidewire. Covidien Palindrome™ heparin-coated catheters were used for permanent placements, while Bard Niagara™ Vas-Cath or Slim-Cath catheters were used for temporary placements. Standard CVC care throughout the study period included using aseptic techniques during CVC and exit-site manipulations, Tegaderm™ dressings for exit-sites, and heparin lock solutions for interdialytic periods. Also, Polysporin^®^ Triple was applied to CVC exit-sites for the first three dressing changes post-insertion. The new CR-infection prevention measures introduced at the midpoint of the study period included masking (patient and nurse) during exit-site care, using 2%-chlorhexidine/70%-alcohol antiseptic instead of povidone-iodine to clean CVC hubs and exit-sites, and administering cefazolin prophylaxis for CVC insertion. There was also an overall effort to reduce the use of temporary CVCs for chronic HD starts.

### Data collection

Information on patient characteristics, HD history, CVC details, and CR-infections was collected from electronic Renal Program databases. To be included, patients had to have received acute or chronic HD for at least 2 weeks during the study period. Data were collected starting from January 1, 2011, for patients already active in the HD program or later for those who joined the program after. Data were collected to March 31, 2014, or sooner for patients who left the HD program due to renal recovery or transplant, switching to peritoneal dialysis, transferring to another location for HD, withdrawing care, or loss of life. Patient characteristics, including age, sex, etiology of kidney disease, dialysis start date, and comorbidities of diabetes or hypertension, were collected. Each CVC was documented according to the type (i.e. temporary-uncuffed or permanent-cuffed), location, and insertion and removal dates. Total CVC days and the relative proportion of temporary and permanent CVCs in use were determined quarterly.

### Infections

Episodes of infection were retrospectively identified in a quality assurance database of CR-ESIs and CR-BSIs that had been documented in real-time by designated personnel in the HD program. The culture date, microbiology, time since CVC insertion, and time to CVC removal were collected for all infections. A CR-ESI required local signs or symptoms of infection (i.e. at least two of redness, tenderness, or pus), a positive culture, and treatment with topical or systemic antimicrobials. A subsequent CR-ESI involving the same catheter was considered a new episode if it occurred at least 2 months later. A CR-BSI required systemic signs or symptoms of infection, a positive blood culture with no other apparent source of infection, and treatment with parenteral antimicrobials. All CR-BSIs were considered new episodes unless the same pathogen was cultured within 3 months in a previously infected catheter that was not removed.

### Statistical analysis

Patients, CVCs, and infections were characterized using descriptive statistics, that is, mean ± standard deviation, median [interquartile range], or number (percentage) as appropriate. Trends in characteristics over time were analyzed using the Mann-Kendall trend test. Quarterly infection rates were calculated as the number of infections per 1000 CVC days. Differences in infection rates between time periods were compared using the two-sided Student’s *t*-test. In addition, potential factors associated with infection, including new CR-infection prevention measures and CVC characteristics, were investigated. Patient characteristics were also considered including age, sex, comorbidities, and duration on dialysis. Univariate analyses were performed with two-sided Student’s *t*-tests, Mann-Whitney *U*-tests, Chi-square or Fisher’s exact tests, or incidence rate ratios (IRR) as appropriate. Pearson’s correlations were used to investigate associations over time.

Modeling was performed to describe the rates of CR-ESI and CR-BSI over the 39-month period while considering potential covariates. Various models were tested, including generalized multiple regression with Poisson distribution and multiple linear regression with and without autocorrelated error. Models were inspected for homoscedasticity, stationarity, and normality. Covariates were considered, including the proportion of temporary CVCs, the number of newly inserted CVCs, and new CR-infection prevention measures. Covariates were tested for collinearity using Pearson’s correlations. Only covariates with statistical significance (*p* < 0.05) were retained in the models. The final models were selected for goodness-of-fit based on the root mean square error (RMSE), coefficient of determination (*R*^2^), and adjusted *R*^2^.

Statistical analyses were performed using SAS^®^ software (SAS Institute Inc.), SYSTAT 13 (Systat Software Inc., San Jose, California), and MedCalc Software Ltd. with a significant level (α) of 0.05.

## Results

### Patients and central venous catheters

**Figure 1.**
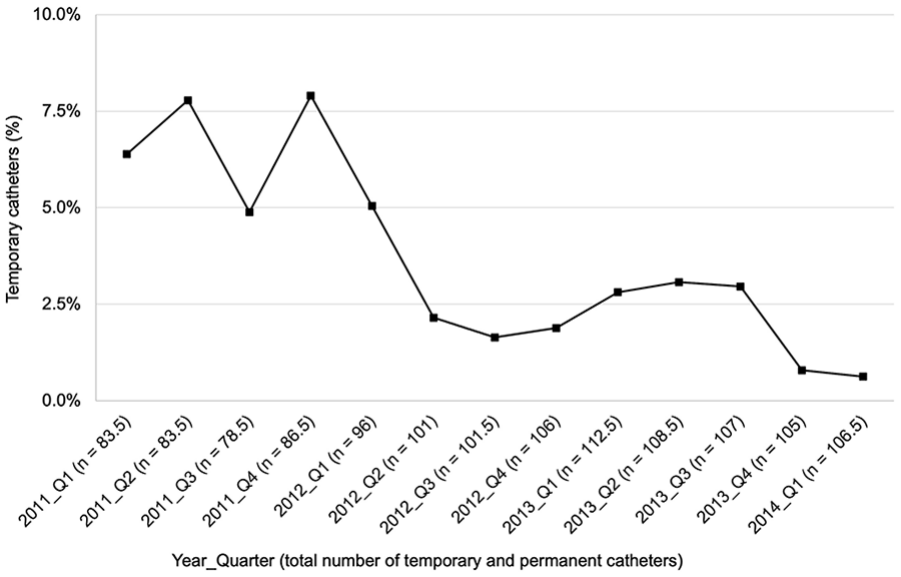
Temporary central venous catheter use over time.

Two-hundred and sixty-seven patients and 499 CVCs representing 114,825 CVC days were captured during the study period. The patient and CVC characteristics are detailed in Tables 1 and 2, respectively. Most CVCs were permanent (78.2%, 390/499), with a significant downward trend in the use of temporary CVCs over time (Figure 1, *p* = 0.004). The proportion of temporary CVCs in place at any given time declined from an average of 5.7% in the first half of the study to 2.0% in the second half. Most CVCs were inserted in the right (77.4%, 386/499) versus the left (17.2%, 86/499) internal jugular vein, and only 1.2% (6/499) were located in the femoral vein.

**Table 1.** Patient characteristics (*n* = 267).

Age (years)^a^	62.9 ± 15.4
Sex	
Male	131 (49.1%)
Female	117 (43.8%)
Undocumented	19 (7.1%)
Comorbidities	
Hypertension	164 (61.4%)
Diabetes	145 (54.3%)
Etiology of kidney disease	
Diabetes	122 (45.7%)
Tubulointerstitial disease	49 (18.4%)
Glomerulonephritis	37 (13.9%)
Hypertension/renovascular disease	33 (12.4%)
Polycystic kidney disease	7 (2.6%)
Reflux	3 (1.1%)
Other	42 (15.7%)
Undocumented	20 (7.5%)
Duration on any type of dialysis (months)	16 [3, 42]
Duration of data collection (months)	9 [2, 25]
Reason for discontinuation of data collection	
End of study	11 (41.6%)
Removed from HD^b^	54 (20.2%)
Transferred to another location for HD	54 (20.2%)
Loss of life	48 (18.0%)

Reported as number (%), mean ± standard deviation, or median [interquartile range].

a Start of study.

b Renal recovery or transplant, switched to peritoneal dialysis, transferred to another hemodialysis unit, care withdrawn.

**Table 2.** Central venous catheters (*n* = 499 in 267 patients).

Type	
Permanent (cuffed)	390 (78.2%)
Temporary (uncuffed)	109 (21.8%)
Time in place (months)	
Permanent	7 [3, 18]
Temporary	1 [0.5, 1]
Location	
Right internal jugular vein	386 (77.4%)
Left internal jugular vein	86 (17.2%)
Other^a^	27 (5.4%)
Insertion time	
Prior to study	78 (15.6%)
During study	421 (84.4%)
Status	
Removed and replaced during study	232 (46.5%)
In place at end of study	102 (20.4%)
Other^b^	165 (33.1%)

Reported as number (%) or median [interquartile range].

a External jugular vein, femoral vein, or subclavian vein.

b Renal recovery or transplant, switched to peritoneal dialysis, transferred to another hemodialysis unit, care withdrawn, loss of life.

### Catheter-related infections

During the 39-month study period, 113 CR-ESIs involving 61 patients and 85 CVCs were identified (Table 3). The overall rate of CR-ESI was 0.98 per 1000 CVC days. Most were monomicrobial infections (85.8%, 97/113), with gram-positive bacteria, gram-negative bacteria, and yeast accounting for 81.2% (108/133), 12.8% (17/133), and 1.5% (2/133) of the pathogens, respectively. The most common organisms were *Staphylococcus aureus* (42.1%, 56/133), coagulase-negative staphylococci (24.8%, 33/133), *Corynebacterium* spp. (9.0%, 9/133), *Pseudomonas aeruginosa* (6.0%, 8/133), *Serratia marcescens* (3.8%, 5/133), and *Streptococcus* spp. (3.8%, 5/133).

**Table 3.** Catheter-related exit-site and bloodstream infections.

CR-infections	Permanent CVCs (*n* = 390)	Temporary CVCs (*n* = 109)
CR-ESIs (*n*)	112	1
CR-ESI rate (per 1000 CVC days)	1.01	0.25
Time from CVC insertion to CR-ESI (months)	8.0 [3.8, 17.0]	4
CVCs involved in one/multiple CR-ESIs (*n*)	63/21	1
CR-BSIs (*n*)	59	5
CR-BSI rate (per 1000 CVC days)	0.53	1.25
Time from CVC insertion to CR-BSI (months)	9.0 [5.0, 17.5]	2.0 [1.0, 3.0]
CVCs involved in one/multiple CR-BSIs (*n*)	37/10	3/1

Reported as number or median [interquartile range]. BSI: bloodstream infection, CR: catheter-related, CVC: central venous catheter, ESI: exit-site infection.

During the study period, 64 CR-BSIs involving 38 patients and 51 CVCs were identified (Table 3). The overall rate of CR-BSI was 0.56 per 1000 CVC days. There was only one polymicrobial infection. Most pathogens were gram-positive bacteria (83.1%, 54/65), including *S. aureus* (53.8%, 35/65), coagulase-negative staphylococci (16.9%, 11/65), and *Streptococcus* spp. (4.5%, 3/65). Gram-negative CR-BSIs were less common (15.4%, 10/65), with *P. aeruginosa* and *S. marcescens* accounting for 6.2% (4/65) and 3.1% (2/65) of the organisms, respectively. Almost one-third of CR-BSIs (32.8%, 21/64) were preceded by a CR-ESI within 3 months. Most cases (85.7%, 18/21) were caused by the same pathogen, usually *S. aureus* (16/18).

### Factors associated with infections

Infection rates were significantly lower after the new CR-infection prevention measures were introduced (1.28 vs 0.73 CR-ESIs per 1000 CVC days (*p* = 0.003) and 0.72 vs 0.50 CR-BSIs per 1000 CVC days (*p* = 0.05)). The CR-BSI rates associated with temporary and permanent CVCs were 1.25 and 0.53 per 1000 CVC days, respectively (IRR = 0.42, *p* = 0.1). There was a strong correlation between the declining CR-BSI rates and the declining use of temporary CVCs over time (Figure 1, rho = 0.73, *p* = 0.005). The CR-BSI rates associated with CVCs located in the left versus right internal jugular vein were 0.82 versus 0.49 per 1000 CVC days, respectively (IRR = 0.59, *p* = 0.09). No infections were associated with CVCs located in the lower extremities. Compared to those without a documented infection during the study period (n = 188), patients with an infection (n = 79) tended to be on dialysis longer (41 [25.5, 64.5] vs 8 [2.0, 27.5] months, *p* < 0.001) and had more CVCs (2.8 ± 1.5 vs 1.5 ± 0.7, *p* < 0.001).

### Modeled infection rates over time

The rates of CR-ESI and CR-BSI were best described by multiple linear regression with autocorrelated error. All homoscedasticity and normality assumptions were satisfied. Covariates for infection were tested but did not significantly improve the models. The proportion of temporary CVCs was not incorporated into the models as it was strongly correlated with time (*r* = −0.83, *p* < 0.001).

**Figure 2.**
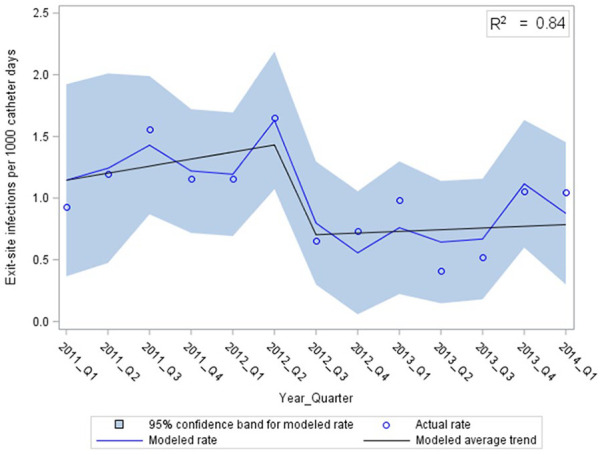
Catheter-related exit-site infections over time, modeled using multiple linear regression with autocorrelated error (RMSE = 0.19).

**Figure 3.**
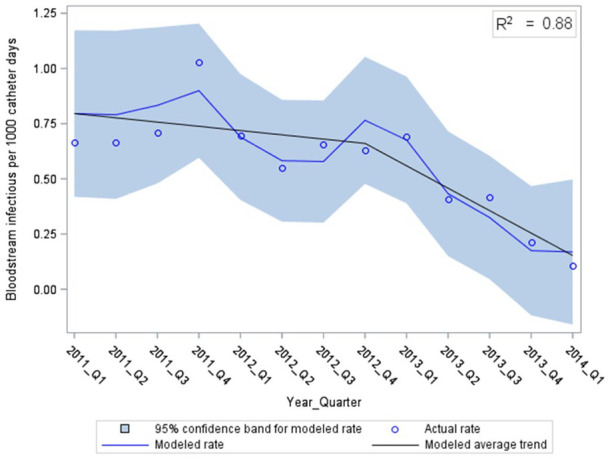
Catheter-related bloodstream infections over time, modeled using multiple linear regression with autocorrelated error (RMSE = 0.11).

The final models describing the rates of CR-ESI and CR-BSI are depicted in Figures 2 and 3, respectively. There was an abrupt and significant drop in CR-ESIs at the midpoint of the study with no further decline (Figure 2, *p* < 0.01). This corresponded to CR-ESI rates of 1.28 compared to 0.73 per 1000 CVC days before and after the new CR-infection prevention measures, respectively (*p* = 0.003). Of note, the rates of CR-ESI appeared to increase in the last two quarters of the study. The rate of CR-BSI decreased throughout the study period, with a more rapid decline starting the quarter after the new CR-infection prevention measures were introduced (Figure 3, *p* < 0.01). The CR-BSI rates were 0.70 per 1000 CVC days before and 0.37 per 1000 CVC days after (*p* = 0.007).

## Discussion

The current study describes a significant reduction in CR-infections over 39 months in patients receiving HD. Notably, the decline coincided with the introduction of new CR-infection prevention measures at the midpoint of the study. The measures included masking during exit-site care, using chlorhexidine-alcohol versus povidone-iodine antiseptic, administering cefazolin prophylaxis with CVC insertions, and reducing temporary CVC use for chronic HD starts. They were selected based on feasibility and evidence available at the time. The use of chlorhexidine-alcohol was supported by strong evidence of superiority over povidone-iodine.^11,23,25–27^ Guidelines did not recommend the routine use of antimicrobial prophylaxis during CVC insertion due to resistance concerns and limited evidence of benefit.^10,11^ However, a study by Huddam et al. showed a reduction in CR-infections when 1 g of cefazolin was administered pre-insertion.^24^ Also, a survey by Smyth et al. published in 2019 revealed that 21% of HD units used antimicrobial prophylaxis, usually a first-generation cephalosporin.^28^ However, due to limited evidence, the routine use of antimicrobial prophylaxis for HD CVC insertions was discontinued at our site. The effort to reduce temporary CVC use was based on substantial evidence of higher infection rates for temporary compared to permanent CVCs.^10^ Likewise, we found a higher rate of CR-BSI in temporary CVCs.

We observed an abrupt drop in CR-ESIs after the new CR-infection prevention measures were introduced, with no further decline. This corresponded to an overall 43% reduction in the rate of CR-ESI. However, there was an increase in CR-ESIs in the last two quarters of the study, which may have signaled weakened compliance with infection prevention measures. The rate of CR-BSI declined steadily throughout the study but more rapidly starting the quarter after the new CR-infection prevention measures were introduced (*p* < 0.01). Notably, the decline in the rate of CR-BSI during the study was strongly correlated with the declining temporary CVC use over time (rho = 0.73, *p* = 0.005).

The rates of CR-infection in the current study were comparable to the values in the literature. Our overall CR-BSI rate of 0.56 per 1000 CVC days was comparable to the range of 0.26–1.86 per 1000 CVC days reported by other studies in North America, Australia, and Europe during a similar time frame.^7,8,12,15–19^ Of note, the rate of CR-BSI in the last quarter of our study, 0.1 per 1000 CVC days, was relatively low. Although there is less published data on CR-ESIs, our rate of 0.98 per 1000 CVC days was comparable to the 0.46–0.80 per 1000 CVC days observed by others.^7,12^ Differences in rates may be explained by variability in patient characteristics, CVC type, and infection control measures. Also, it is important to consider that the criteria used to define CR-ESI and CR-BSI in the literature are inconsistent. The depth of catheter data collected in the current study was novel and prevented over-estimation of infection rates. More specifically, our data allowed for the identification of infection relapses when the same pathogen was cultured from a catheter that was not removed.

The microbiological etiology of CR-infections in our study was consistent with older reports in Canada.^12,29^ Over 80% of infections were caused by gram-positive bacteria, primarily skin flora, with *S. aureus* and coagulase-negative staphylococci accounting for 46% and 22% of cases, respectively. The microbiology was similar between CR-BSIs and CR-ESIs, again demonstrating the important connection between the two infection types. These data help direct the selection of appropriate empirical therapy, especially since prompt antimicrobial therapy significantly improves treatment outcomes.

In our study, potential factors associated with infection were also explored. Consistent with other studies, we observed a higher CR-BSI rate for CVCs located in the left versus right internal jugular vein, although the mechanism remains unclear.^10,30,31^ In addition, patients who developed infection tended to be on dialysis longer and, therefore, “at risk” for longer. Those with an infection also had more CVCs since infected catheters are often replaced. Finally, almost one-third of CR-BSIs were preceded by a CR-ESI within 3 months. Most of these cases were caused by *S. aureus*, known to produce biofilms on indwelling devices. Taylor et al. also described a significantly increased BSI risk following HD access site infection (Odds Ratio = 4.36, *p* = 0.002)^2^. Overall, CR-ESIs remain an understudied risk factor for CR-BSIs. Our findings highlight the significance of optimal exit-site care, effective CR-ESI treatment, and ongoing surveillance.

One limitation of the current study is that data were collected retrospectively. As such, it is possible there were unidentified biases or confounders. For instance, the use of immunosuppressive agents was not available in our data; however, their use significantly increases infection risk. Also, the nature of our data did not allow for the determination of causation, only association. Finally, it is important to consider the geographical context and time period when interpreting the study results. Even so, these data are enlightening and fill a substantial gap in the study of CR-infections in patients receiving HD, particularly for CR-ESIs. Moving forward, further surveillance is necessary to maintain the applicability of this initial work, especially given the evolving nature of infection control measures. This is particularly true given notable changes in infection control following the COVID-19 pandemic.

## Conclusions

In summary, the current study describes a significant decline in CR-infections over 39 months in patients receiving HD. More specifically, there was an abrupt drop in CR-ESIs after new CR-infection prevention measures were introduced. However, by the end of the study, ESI rates began to rise. Also, during the study, there was a steady decline in CR-BSIs, which was highly correlated with a concurrent decrease in temporary CVC use. Notably, ESIs preceded one-third of BSIs, primarily caused by the same pathogen. These findings emphasize the need for ongoing surveillance, reinforcement of CR-infection prevention measures, and optimal CR-ESI prevention and treatment to reduce the risk of CR-BSIs.
